# Micropeptides translated from putative long non-coding RNAs: Micropeptides translated from lncRNAs

**DOI:** 10.3724/abbs.2022010

**Published:** 2022-02-23

**Authors:** Junhong Li, Lei Qu, Lingjie Sang, Xinyu Wu, Anlan Jiang, Jian Liu, Aifu Lin

**Affiliations:** 1 MOE Laboratory of Biosystem Homeostasis and Protection College of Life Sciences Zhejiang University Hangzhou 310058 China; 2 Zhejiang University-University of Edinburgh Institute (ZJU-UoE Institute) Zhejiang University School of Medicine International Campus Zhejiang University Haining 314400 China; 3 Hangzhou Cancer Institution Affiliated Hangzhou Cancer Hospital Zhejiang University School of Medicine Zhejiang University Hangzhou 310002 China; 4 International Institutes of Medicine the 4th Affiliated Hospital of Zhejiang University School of Medicine Yiwu 322000 China; 5 Cancer Center Zhejiang University Hangzhou 310058 China; 6 Breast Center of the First Affiliated Hospital School of Medicine Zhejiang University Hangzhou 310003 China; 7 Key Laboratory for Cell and Gene Engineering of Zhejiang Province Hangzhou 310058 China

**Keywords:** long non-coding RNA (lncRNA), small open reading frame (sORF), micropeptide, coding-potential

## Abstract

Long non-coding RNAs (lncRNAs) transcribed in mammals and eukaryotes were thought to have no protein coding capability. However, recent studies have suggested that plenty of lncRNAs are mis-annotated and virtually contain coding sequences which are translated into *bona fide* functional peptides by ribosomal machinery, and these functional peptides are called micropeptides or small peptides. Here we review the rapidly advancing field of micropeptides translated from putative lncRNAs, describe the strategies for their identification, and elucidate their critical roles in many fundamental biological processes. We also discuss the prospects of research in micropeptides and the potential applications of micropeptides.

## Introduction

As a subclass of transcripts, long non-coding RNAs (lncRNAs) were firstly characterized by a wide-range sequencing of full-length cDNA libraries [1]. LncRNAs are a group of autonomously transcribed RNAs with a length longer than 200 nucleotides (nt), and their functions lack understanding [ 2– 6]. LncRNAs are transcribed in an mRNA-like way by RNA polymerase II, undergo 5′ capping, polyadenylation, and splicing [ 7, 8]. Previous studies have shown that lncRNAs are emerging as critical regulators in different cellular homeostasis and gene expression regulation, rather than protein-coding capability [ 9– 13].

Transcriptome analysis serves as a foundation to understand the coding and non-coding regions of the genome. The GENCODE comprehensive high-throughput RNA sequencing has identified a large number of unannotated long non-coding loci by a series of computational analyses, manual annotations, and experimental validations [ 14– 17]. However, many small open reading frames (sORFs) in the long non-coding transcripts are involved in protein translation, which were disregarded or mis-annotated by genome annotations according to the rule for protein synthesis that an ORF should contain codons of at least 100 amino acids in length from the start codon to the stop codon [18]. Thus, the main challenge in the annotation of sORFs in the long non-coding transcripts is to identify *bona fide* functional peptides and exclude those real “transcription noises” that are not translated. The advances in RNA sequencing technology, such as ribosome profiling, have led to the profound investigation and comprehension of the coding potential of lncRNAs [ 19– 24].

This review presents an overview of the approaches developed and their applications in the systematic annotation of sORFs in long non-coding transcripts, showing that the translation of mRNAs is more extensive than we ever thought. We systematically discuss how these innovative techniques are utilized to spot *bona fide* functional peptides which are bioactive in multiple cellular processes. We further discuss both the advantages and disadvantages of all these strategies in identifying the coding potential of lncRNAs. Moreover, we also discuss some well-known micropeptides and their functions, and provide perspectives about the prospect of micropeptides.

## Identification of Potential Protein-coding sORFs from Putative lncRNAs

Deciphering the genetic information encoded by the genome precisely is still a significant challenge. However, in recent years, fine-tuned bioinformatics methods and experimental or sequencing techniques have been significantly improved and successfully implemented to predict and analyze the potential sequences that were previously considered to be non-coding sequences. In this section, we discuss the approaches for the identification of the potential protein-coding sORFs from putative lncRNAs.

### Ribosome profiling

**Figure 1:**
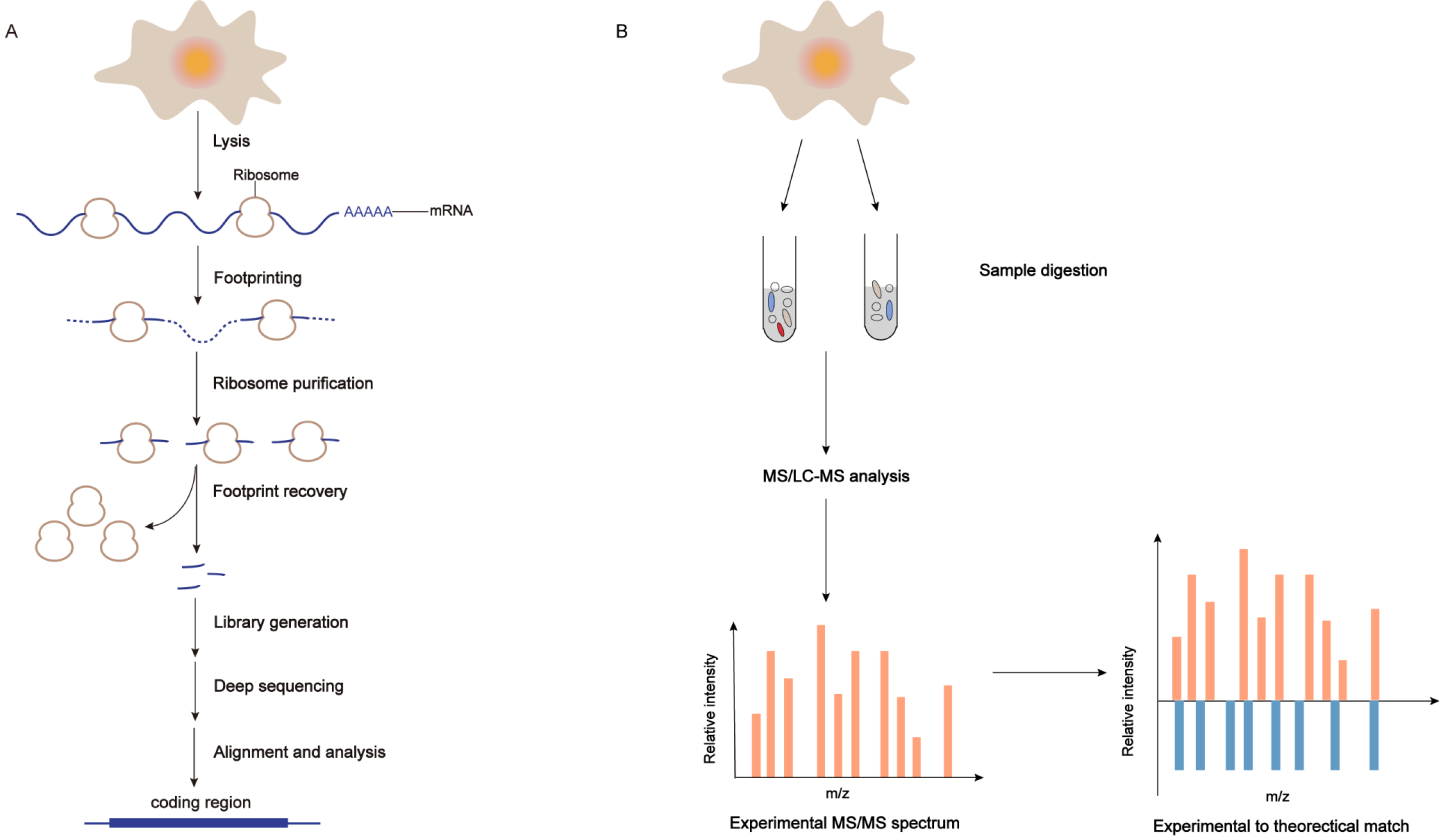
Figure1 Techniques for the identification of micropeptides from putative lncRNAs Some techniques have emerged or developed as experimental methods to verify the coding potential of sORFs from putative lncRNAs. (A) The workflow of simplified ribosome profiling. In this technique, we can measure the comprehensive translation information by deep sequencing of ribosome-protected fragments (RPFs) after manually disrupting the active translation. (B) Mass spectrometry identifies micropeptides encoded by sORFs of lncRNAs by matching the experimental MS spectra with predicted spectra from a reference or custom database.

Ribosome profiling, as a rising RNA sequencing technology, has dramatically contributed to the field of translatome in the past decade. It gives us a chance to reveal the complexity of genetic information [ 20– 24]. Relative to traditional RNA sequencing which aims at complete RNA sequences, ribosome profiling has its preference for ribosome-protected fragments (RPFs). Generally speaking, ribosome profiling is a sensitive technique that provides a genome-wide snapshot of the region actively translated in the cells and relies on the fundamental principle that translating ribosomes can protect ~30-nt long RNA sequence from digestion by nucleases [ 19, 22– 28]. In addition, as the ribosome scans the sequence one codon at a time, the reads remain a precise three-nucleotide periodicity and show a single nucleotide resolution. Cytoplasmic lysates are prepared with the addition of translation inhibitors and the resultant mRNA-ribosome complexes are treated with nucleases to produce RPFs. These RPFs can be isolated and purified for further sequencing ( Figure 1A). Therefore, we can get the integrated information of translation and the rates of protein synthesis in the actively translated regions [ 19, 20, 29].

With this technique, many putative lncRNAs were identified to have coding potential [20]. Ribosome profiling is a practical method to evaluate the coding potential of the regions of interest. However, the occupation of elongated ribosomes by some sequences does not necessarily mean that active translation happens or protein synthesis occurs [30]. There is a linear correlation between the level of mRNA and ribosome signal, while the positive correlation is not suitable for lncRNAs [23]. The ribosome signal of lncRNAs suggests that they may be associated with the ribosome machinery to regulate canonical translation. Meanwhile, RPFs reads do not equal to actively translated fragments. Pseudogenes have been found to bind with ribosomes, indicating that they also contribute to the partial reads of RPFs [31]. The translation of proteins is not a simple process of reading codons from the start codon to the stop codon, it also includes the accurate complex assembly and regulatory mechanism [ 32– 34].

Hence, ribosome occupancy cannot be the only metric to identify whether an lncRNA has a coding capacity. Other techniques or metrics have been developed to more accurately identify actively-translated sORFs. For example, a metric named ribosome release score (RRS) is helpful to clarify whether the transcripts are coding or non-coding. The method is developed according to the rule for translation that mRNAs can disassociate from ribosome when a stop codon is reached. Hence the method has higher sensitivity by detecting the termination of translation at the end of an ORF. In addition, more recent approaches that take the entire 3′ untranslated region (UTR) into consideration can produce more accurate results for coding transcripts compared to RRS [ 30, 35]. With the help of RRS, one-third of the lncRNAs were proved to be translated in murine embryonic stem (ES) cells [35].

Moreover, lots of algorithms and methods, such as ORF-RATER [36], Proteoformer [37], ORFscore [38], FLOSS [26], and TOC [39], have been developed and used to analyze and manage the raw data generated from ribosome profiling. In addition, a significant number of databases were created in succession based on the research of ribosome profiling and its production, including RPFdb [40], SmProt [41], TISdb [42], GWIPS-viz [43], and sORFs.org [44]. These databases have been proven to be invaluable for the research in micropeptides. In sum, many techniques have been developed to identify the coding capacity of putative lncRNAs.

### Mass spectrometry

Mass spectrometry (MS)-based proteomics is gold standard for the detection and characterization of proteins. MS is able to detect the expressions proteins and polypeptides as well as their interactions indirectly through measuring the ionized polypeptides or proteins in a gaseous state [45] ( Figure 1B). Therefore, this technique evaluates the coding potential of putative lncRNAs and validates the translation of microproteins from sORFs.

To get a robust result for identifying micropeptides, MS proteomics, referred to as proteogenomics, is usually used in combination with genomic techniques like RNA sequencing [ 46– 48]. Researchers have characterized lots of micropeptides by utilizing proteogenomics or MS-based methods in some cell lines [ 49, 50]. Bánfai *et al*. [49] used tandem mass spectrometry (MS/MS) data mapping sORFs and compared polyA+ and polyA– RNA-seq data generated by ENCODE in the cell lines of K562 and GM12878. They developed a machine-learning algorithm RuleFit3 and predicted peptides from lncRNAs in these two cell lines. Unexpectedly, only a few lncRNAs were found to have protein-coding ability, which might result from the fact that they set 23 amino acids as a threshold, possibly causing some missed detections [49]. Slavoff *et al*. [50] combined peptidomics with RNA-Sequencing to characterize sORFs in K562 cells by utilizing proteogenomics. Based on the annotated translatome from the human genome (RefSeq), they built a custom database which included all sequences with coding-potential for micropeptides greater than eight amino acids. They then utilized liquid chromatography-tandem with mass spectrometry (LC-MS/MS) to detect the usually missed sORF-encoded polypeptides (SEPs) and match their proteomics data with a custom sequencing database. Using the indicated technique, they finally identified 86 previously uncharacterized polypeptides in K562 cells [50]. In general, MS-based methods have shown their superiority in the detection and identification of novel micropeptides.

It should be noted that micropeptides are frequently lost in the prepared samples, leading to the unsatisfactory MS detection in many cases. What is worse, even with careful and special treatment to protect micropeptides from unnecessary loss, small proteins are still not easy to identify by MS due to the difficulties in the detection of the fragmented products [51].

Additionally, the introduction of specific proteases for digestion during the sample preparation process does not guarantee that microproteins can be fragmented efficiently and the signatures are strong enough to be detected. Furthermore, the concentration issue of small peptides should not be neglected, since at a low concentration, peptides are hard to be detected due to the difficulties in distinguishing the signals of target polypeptides from the noises [ 51, 52].

In sum, MS-based proteomics is a powerful method that benefits the discovery and identification of uncharacterized small proteins. Generally, identification of the micropeptides produced by MS can provide credible evidence for the presence of SEPs. However, the missing of SEPs does not mean its absence in translation in principle. So in the future we need to take advantage of interdisciplinary research between computational science and experimental research to characterize small peptides precisely and efficiently.

### Computational approaches

Even though some lncRNAs have been recognized to produce micropeptides, how to identify them is still a big challenge. Computational approaches that distinguish coding and non-coding sequence are intended to evaluate the characteristics of RNA sequence and locus information of transcripts of interest. The evaluation is mainly based on intrinsic sequence characteristics, including nucleotide composition, intron splice sites, ORF length, sequence homology to known protein sequences, sequence conservation and substitution ratio, and secondary structure [ 53– 57]. Although traditional computational approaches that do not utilize ribosome profiling or MS data have been successfully used to evaluate the coding potential of some lncRNAs, they fail to validate microproteins translated from sORFs of lncRNA [53]. However, things were changed when computational approaches were utilized to integrate the ribosome profiling data with MS data. Application of the indicated computational approaches that greatly rely on experimental data and get rid of the restriction of transcript length enables the detection of the reliable translation of sORFs of lncRNAs successfully.

Some algorithms have been developed to evaluate the coding potential of sORFs from lncRNAs. For example, translated ORF classifier (TOC) evaluates the coding potential of sORFs using four features based on ribosome profiling data [58]. ORF-RATER is an algorithm based on the linear regression of RPF coverage with harringtonine and lactimidomycin that induce translation stalling at the start codon [36]. Coding Region Identification Tool Invoking Comparative Analysis (CRITICA) is a computer program that characterizes coding sORFs by combining comparative analyses and the statistics of coding sequences [59]. In contrast, periodicity-based approaches incline to get sufficient ribosome coverage. For instance, ORFscore which reflects the significance of three-nucleotide periodicity detected 190 sORFs coding for peptides of 20–100 amino acids in length during the analysis of Ribo-seq data from zebrafish embryos [38]. Phylogenetic codon substitution frequency (PhyloCSF) analysis is a powerful computation approach recommended by University of California Santa Cruz (UCSC) Genome Browser, which is easily accessible to most researchers. It examines the evolutionary signatures of nucleotide sequence alignment to determine the likelihood of a conserved protein-coding region based on a formal statistical comparison of phylogenetic codon models [60]. Computational approaches have exhibited their powerful function in the identification of thousands of sORFs, however it is vital to combine with other experimental methods to insure their reasonability, efficiency, and reliability.

## Characterization of Functional Micropeptides from sORFs of Putative lncRNAs

Recent work has identified thousands of additional components of the proteome, which are micropeptides translated from sORFs in the non-coding region. Many sORFs have been examined for their coding potential, and their productions are able to be regarded as authentic proteins playing necessary roles in aspects of biological processes in living organisms [ 61– 63]. However, translated polypeptides with evolutional conservation do not suggest that they have a virtual biological function. Therefore, experimental validation of their physiological roles is necessary [51].

### Validation of translation of sORFs from putative lncRNAs

A traditional way to validate the translation of sORFs is to determine their *in vivo* translation. Another way is to utilize antibodies against the target peptides to help us examine the endogenous expression of small peptides, since antibodies can specifically recognize proteins. However, it is still a challenge to generate specific and effective antibodies against micropeptides in some cases. One reason is that these small peptides have low antigenicity due to their short sequences [51]. Another question that should be addressed is that the structure of micropeptides may contain transmembrane domains, which largely restricts the choice of epitope design [ 64– 67]. Additionally, other techniques ( *e*. *g*., western blot and immunohistochemical assays) heavily rely on the specificity and affinity of antibodies [51].

**Figure 2:**
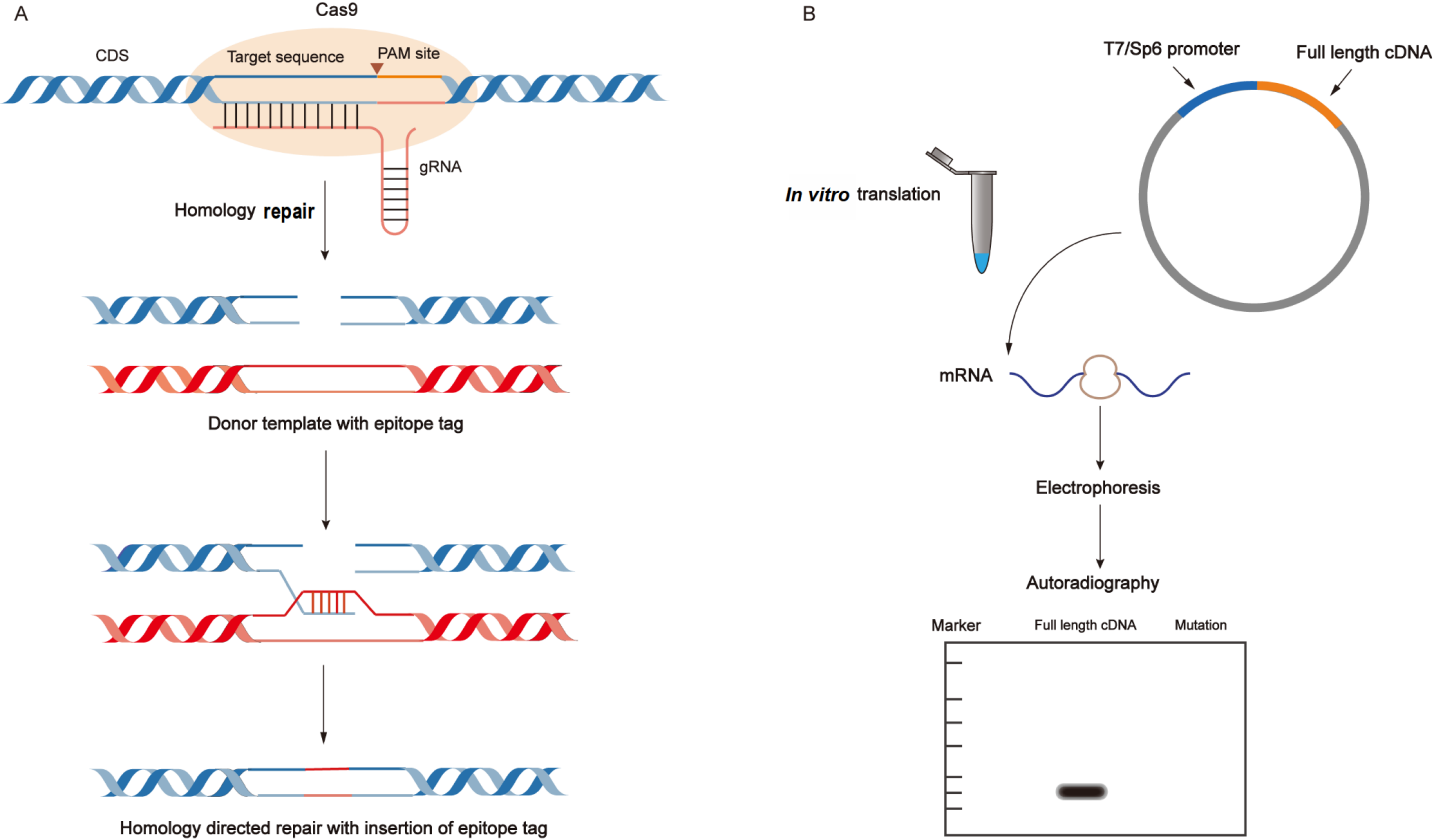
Figure2 Methods for the verification of coding potential of sORFs from putative lncRNAs (A) CRISPR-CAS9-mediated gene editing helps to knock in epitope tag into the endogenous locus of putative micropeptides. Then, we can detect the expressions of the fusion proteins by western blot analysis and immunostaining. (B) In vitro translation is also an alternative method to confirm the coding potential of sORFs. Firstly, the full length cDNA should be cloned into a vector with a phage polymerase promoter. Secondly, the expression of the vector is validated by the protein synthesis in the environment of protein-free and the presence of 35S-methionine. Thirdly, the protein products are subject to gel electrophoresis and autoradiography. Therefore, we can determine the molecular weight and expression of a protein. A negative control, a frame-shift mutation, is necessary for this experiment.

Because some antibodies may be ineffective, other techniques are required to evaluate the potential coding micropeptides effectively. For example, epitope tagging is a good alternative to detect small peptides. By using CRISPR (Clustered Regularly Interspaced Short Palindromic Repeats)/Cas9 (CRISPR-Associated Protein 9)-mediated gene-editing technologies, epitope tag sequences can be inserted into the endogenous locus of micropeptides that we are interested in ( Figure 2A) [68]. Fusion protein created by CRISPR/Cas9 can be detected by western blot analysis and immunoprecipitation. However, the CRISPR/Cas9 technique should avoid the impact on the local signal of micropeptides during insertion of an epitope tag into the genome locus [69]. Additionally, although epitope tagging has been regarded as an effective tool that provides convincing evidence for endogenous expression of micropeptides, its efficiency depends on the cell lines used [ 51, 70].

*In vitro* translation assay is another common method to identify the coding potential of sORF [ 71, 72]. During translation assay, full-length double-strand cDNAs encoding putative micropeptides are integrated into a vector that includes a phage polymerase promoter (T7 or SP6) ( Figure 2B). The translation of proteins can validate the expression of the vector in a protein-free environment and in the presence of ^35^S-methionine. The generated proteins are analyzed by gel electrophoresis and autoradiography which can distinguish the coding transcripts from the non-coding transcripts by detecting the synthesis of ^35^S-methionine-labeled peptides. Although this technique provides enough evidence that an sORF is translated *in vitro*, additional experiments need to be performed to validate the expressions of micropeptides. Introduction of a frameshift mutation in sORFs of putative micropeptides, which destructs the ORF in-frame and abolishes the expression of micropeptide, is often used as a negative control to further verify the result [51].

### Biological function of micropeptides from putative lncRNAs

Some micropeptides from putative lncRNAs have been carefully identified by different experimental verifications. The results have shed light on the crucial roles of micropeptides in multiple physiological processes. Now the investigations of the roles of micropeptides are very extensive in specific cells or tissues. Therefore, we’ll briefly review the functions of micropeptides in several aspects.

#### Tumor-related micropeptides from putative lncRNAs

##### FORCP

**Figure 3:**
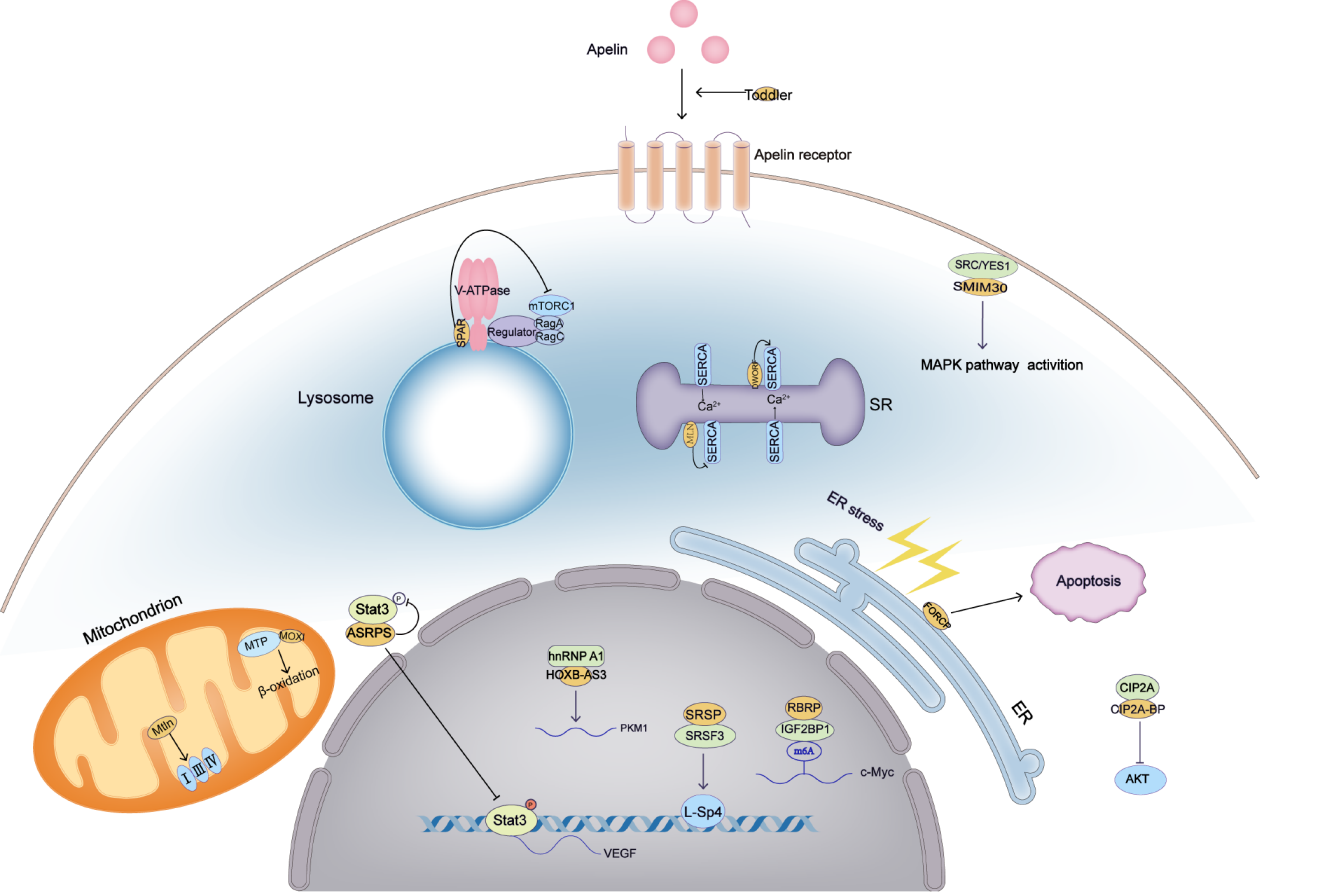
Figure3 Various biological roles of micropeptides encoded by putative lncRNAs These peptides play diverse biological roles in suppressing or promoting tumorigenesis and/or tumor progression, responding to stress, regulating mRNA translation, promoting gastrulation formation, enhancing metabolic homeostasis, regulating muscle generation, and improving muscle performance.

By using PhyloCSF, an algorithm that evaluates the potentiality of a coding sequence through alignment of codon substitution frequency, Li *et al*. [73] found that *LINC00675* has the potential to be translated. Subsequent experiments validated that the well-conserved 240 nt ORF of *LINC00675* is translated into a 97-amino acid small protein, termed FOXA1-regulated conserved small protein (FORCP). The results showed that FORCP is primarily localized on the endoplasmic reticulum (ER) and specifically abundant in well-differentiated colorectal cancer (CRC) cells ( Figure 3). They also discovered that FORCP inhibits basal proliferation and induces apoptosis upon ER stress in well-differentiated CRC cells.

##### SRSP

To discover putative lncRNAs that have the potential to be translated into polypeptides, Meng *et al*. [74] performed ribosome-bound RNA-seq and bioinformatics analysis, and found that lncRNA *LOC90024* encodes a 130-amino acid small protein, termed splicing regulatory small protein (SRSP). They showed that SRSP facilitates colorectal cancer (CRC) initiation and progression. Mechanically, SRSP interacts with serine and arginine rich splicing factor 3 (SRSF3), an mRNA splicing factor [ 75, 76], to regulate mRNA splicing. SRSP enhances the binding of SRSF3 with the exon 3 of transcription factor Sp4, which causes the formation of the oncogenic long Sp4 isoform and represses the generation of non-oncogenic short Sp4 isoform ( Figure 3). Thus, SRSP may be a novel prognostic biomarker and therapeutic target of CRC [74].

##### RBRP

Zhu *et al*. [77] found that *LINC00266-1*, which was previously annotated as an lncRNA in *Homo sapiens*, actually encodes a 76-amino acid micropeptide named “RNA-binding regulatory peptide” (RBRP). Mechanically, RBRP interacts with m6A reader IGF2BP1 and facilitates CRC development by increasing the stability of *c-Myc* mRNA ( Figure 3). Furthermore, high expression of RBRP is correlated with poor prognosis in CRC patients. In short, this study characterized that RBRP, an oncopeptide, binds to m6A reader and affects its recognition on target mRNAs, resulting in turmorigenesis.

##### SMIM30

Pang *et al*. [71] discovered a series of ribosomal protein S6 (RPS6) bound by lncRNAs using RIP-seq. Among these candidates, *LINC00998* encodes a 59-amino acid peptide termed SMIM30. The coding protein, not lncRNA itself, contributes to hepatocellular carcinoma (HCC) tumorigenesis by promoting cell proliferation and migration. In addition, high expression level of SMIM30 was found to be positively associated with a poor survival rate in patients with HCC. The results showed that SMIM30 actively interacts with SRC/YES1, a non-receptor tyrosine kinase, to switch on the mitogen-activated protein kinase (MAPK) pathway ( Figure 3). They revealed the potential of micropeptides as biomarkers for HCC diagnosis and prognosis and new therapeutic targets for HCC.

##### ASRPS

It was reported that lncRNA *LINC00908* has a dual role in different cancers. *LINC00908* is able to inhibit the progression of prostate cancer by sponging miR-483-5p, which represses the expression of testis-specific Y-like protein 5 (TSPYL5) [78], while *LINC00908* promotes HCC progression by enhancing the stability of Sox-4 [79]. A recent study conducted by Wang *et al*. [80] revealed the coding role of *LINC00908* in triple-negative breast cancer (TNBC). They identified a 60-amino acid polypeptide called ASRPS (a small regulatory peptide of STAT3) translated from *LINC00908*. ASRPS expression is expressed at a low level and correlated with poor prognosis in TNBC. They further explored the function of ASRPS in TNBC and found that ASRPS directly binds with the coiled-coil domain (CCD) of STAT3 and impairs its phosphorylation, which results in a low expression level of VEGF ( Figure 3). Therefore, ASRPS functions as a tumor suppressor and can be a potential target for TNBC treatment.

##### HOXB-AS3

LncRNA *HOXB-AS3*, also known as HOXB cluster antisense RNA 3, is a well-known lncRNA with critical roles in different ways in multiple cancers [ 81– 83]. Huang *et al*. [84] discovered that HOXB-AS3 encodes a 53-amino acid small peptide. They further confirmed that the HOXB-AS3 peptide, rather than lncRNA itself, inhibits tumorigenesis by affecting hnRNP A1-dependent PKM splicing and metabolic reprogramming in CRC cells ( Figure 3). The results suggested that low expression of HOXB-AS3 peptide is correlated with a poor survival rate of cancer patients. Overall, the HOXB-AS3 peptide is an anti-tumor micropeptide that represses PKM splicing and cancer metabolism.

##### CIP2A-BP

Using bioinformatics and experimental tools, Guo *et al*. [85] found a 52-amino acid peptide translated from *LINC00665*, a putative lncRNA. The micropeptide was named CIP2A-BP, whose translation level is downregulated by TGF-β treatment in breast cancer cell lines. In addition, downregulated CIP2A-BP was found to be correlated with a poor prognosis and also with tumor invasion and metastasis. Mechanistically, CIP2A-BP directly interacts with CIP2A to increase the activity of protein phosphatase 2A (PP2A), an inhibitor of the PI3K/AKT/NF-κB pathway, ultimately contributing to the expression of a downstream target of the signaling pathway ( Figure 3). In conclusion, they identified a micropeptide encoded by lncRNA, which functions as a tumor repressor.

#### Muscle-related micropeptides from putative lncRNAs

##### MLN

Anderson *et al*. [72] discovered that lncRNA LINC00948, which is a skeletal muscle-specific RNA, encodes a 46-amino acid micropeptide termed myoregulin (MLN). They found that MLN is an inhibitor of sarcoendoplasmic reticulum calcium transport ATPase (SERCA). MLN directly binds with SERCA which has a single transmembrane alpha-helix to impair Ca ^2+^ uptake into the sarcoplasmic reticulum (SR) ( Figure 3). In reverse, depletion of MLN in mice results in enhanced Ca ^2+^ pump activity and exercise performance.

##### DWORF

Using PhyloCSF, Nelson *et al*. [66] discovered an unrecognized ORF of 34 codons in lncRNA *LOC100507537*, which encodes a 34-amino acid micropeptide named DWORF. DWORF locates in the SR membrane and releases the activity of SERCA that is impeded by phospholamban, sarcolipin, and myoregulin ( Figure 3). Thus, DWORF enhances muscle performance by activating calcium pumps, which may provide a possible means for the treatment of heart diseases [66].

##### SPAR

Matsumoto *et al*. [86] identified a novel polypeptide referred to as SPAR. SPAR is conserved between mouse and human and encoded by the lncRNA *LINC00961*. The results suggested that SPAR downregulation promotes the activation of mTORC1 and facilitates muscle regeneration. Mechanistically, SPAR is predominantly located in the endosome/lysosome where it negatively regulates mTORC1 activation by interacting with the lysosomal v-ATPase.

#### Other functional micropeptides from putative lncRNAs

Other studies also reported some micropeptides encoded by putative lncRNAs. Mitoregulin, also known as Mtln, is a 56-amino acid peptide encoded by *LINC00116*. Mtln localizes to the mitochondrial inner transmembrane and enhances respiration efficiency by bolstering super complex assembly [65] ( Figure 3). Makarewich *et al*. [64] reported a conserved micropeptide named MOXI. The peptide plays a critical role in the control of mitochondria metabolism, contributing to fatty acids β-oxidation ( Figure 3). Finally, Pauli and colleagues discovered a zebrafish micropeptide, Toddler, encoded by a transcript previously annotated as non-coding. Toddler directly binds to apelin receptor (APJ) and activates APJ/apelin receptor signaling to promote cell movement in zebrafish [87] ( Figure 3).

## Concluding Remarks and Future Perspectives

Recent studies have gradually discovered and characterized a large number of micropeptides encoded by putative lncRNAs in multiple species. It further reveals the complicacy of gene translation and reminds us to reconsider cautiously about the lncRNAs that were previously annotated as non-coding RNAs. Emerging evidence also indicated that micropeptides translated from putative lncRNA are involved in multiple biological activities, which expands the scope of lncRNA gene expression.

The discovery of micropeptides is heavily dependent on the recent progress of computational approaches and experimental methods, *e*. *g*., ribo-seq [19], proteogenomics [45], and phyloCSF [60]. So, the improvements of existing technologies will be essential for further research in micropeptides. In addition, with regard to bifunctional lncRNAs that have dual roles at the RNA and protein levels, discriminating their coding and non-coding functions is of great significance in some ways. In particular, considering the limited copy number of lncRNAs [ 13, 88], it is attractive to figure out how bifunctional lncRNAs exert and coordinate dual biological functions at such a low expression level.

Although recent findings have unveiled the essential roles of micropeptides encoded by lncRNAs, future research is needed to identify more micropeptides and their functions. Prevail large-scale sequencing uncovered a number of cancer-related genes, especially the mutations of protein-coding genes referred to as the drivers of cancers [ 89– 91]. In addition, comprehensive analyses of driver mutations in non-coding regions have been performed by a couple of studies [ 92– 94]. For example, they focused on the non-coding regions of protein-coding genes instead of the non-coding genes. The latest work has shed light on the mutations of non-coding genes. Cao *et al*. [94] shifted their attention to the splicing sites created by non-coding mutations in cancer. In their work, most of the non-coding mutations can generate new exons in both the coding and the non-coding genes, suggesting the potential importance of non-coding mutations in cancer. Furthermore, the new exons created by non-coding mutations are likely to produce novel proteins and/or micropeptides. Therefore, additional work is needed to explore the non-coding mutations in non-coding genes.

We have briefly reviewed a subset of representative tumor-related micropeptides encoded by lncRNAs, and many of them may be potential prognostic markers and therapeutic targets [ 71, 73, 76, 77, 80, 84, 85]. Additionally, given the small size of micropeptides, high specificity and low cytotoxicity, micropeptides may be used as potential drugs for cancer treatment [95]. For example, recombinant MP31, a micropeptide encoded by the uORF of PTEN, prominently inhibits the formation of brain tumors derived from GBM cells and improves the overall survival in mice [96]. The man-made MOTS-c [97], a mitochondrial-derived peptide, alleviates osteolysis in a mouse model, which exhibits great potential in the treatment of bone erosion. The WT-iRGD peptide [98] based on the design of amino acids 1–10 of Rab22a-NeoF1 could inhibit lung metastasis in the mouse model of osteosarcoma metastasis. Thus, these results strongly support the clinical application of micropeptides.

In summary, gene expression is more complicated than we previously thought, due to the existence of a family of non-coding genes. The discovery of micropeptides makes it more difficult to fully understand the pattern of gene expression. Recent studies have uncovered part of the mystery in micropeptides by well-established research methods. Although thousands of micropeptides have been identified by large-scale screening in multiple species, many of their functions remain unknown. Therefore, future work should focus on elucidating their biological roles and exploring their diverse functions.
